# Supplementary material for: Common infections and neuroimaging markers of dementia in three UK cohort studies
Source: Alzheimers Dement. 2024 Jan 22;20(3):2128–42. doi: 10.1002/alz.13613 (PMC10984486; doi:10.1002/alz.13613)
Supplement: Supplementary file 2 — Supporting Information [file ALZ-20-2128-s005.pdf]

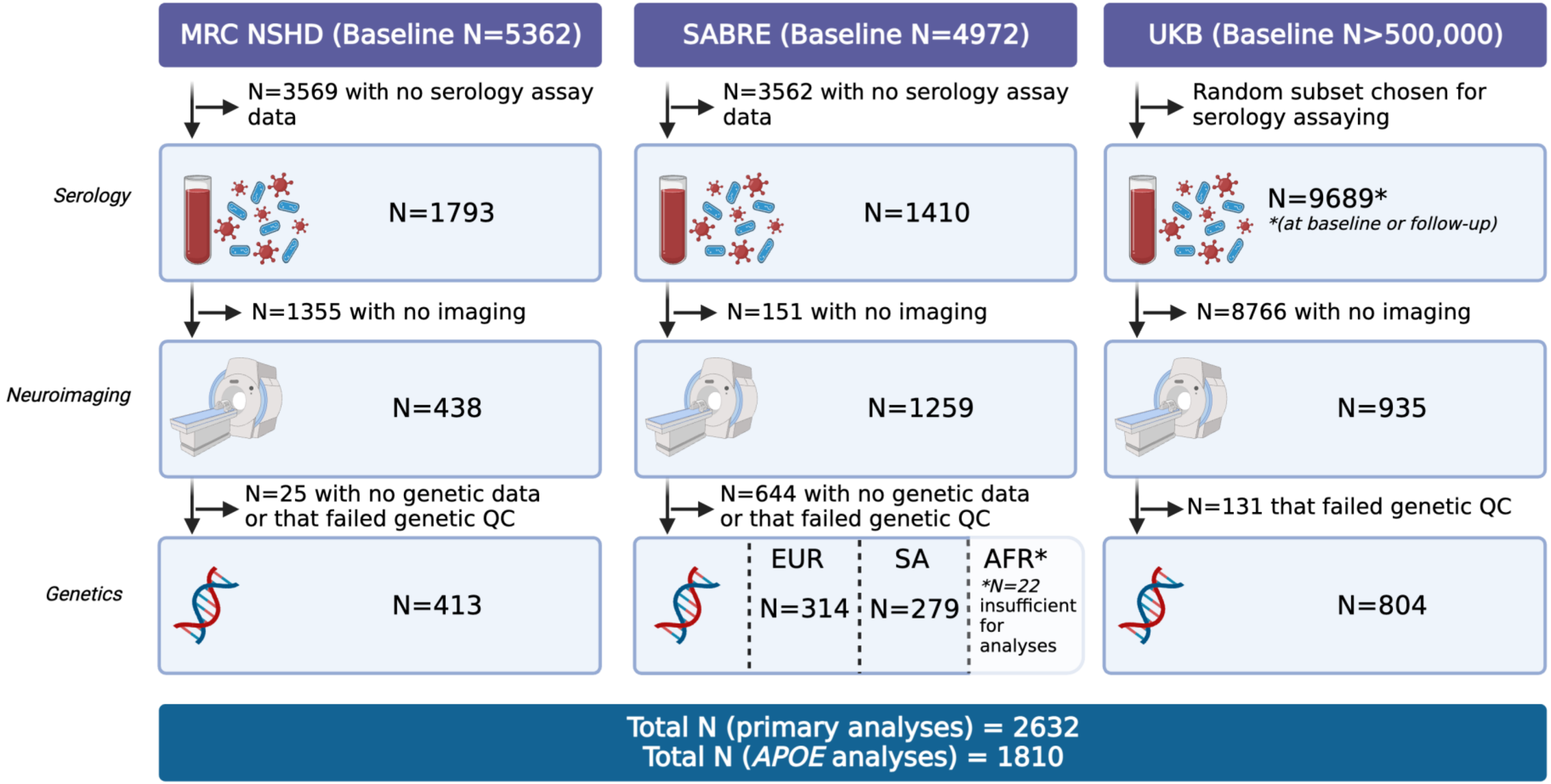

Supplementary Figure 1. Overview of study participants.
Abbreviations: AFR=African Caribbean, *APOE*=Apolipoprotein E genotype, EUR=European, MRC NSHD=Medical Research Council National Survey of Health and Development, SA=South Asian, SABRE=Southall and Brent Revisited, QC=quality control, UKB=UK Biobank.

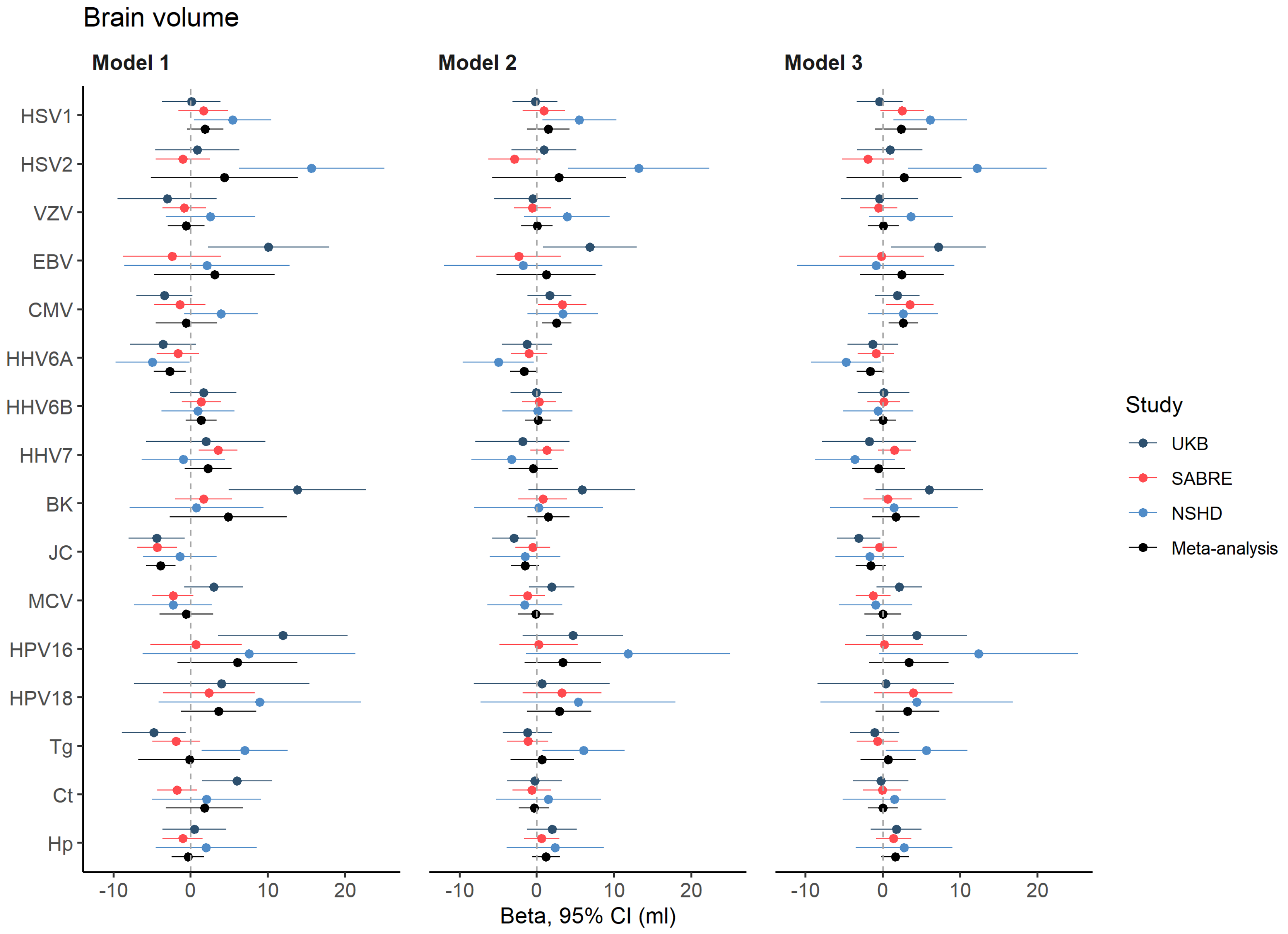

Supplementary Figure 2. Forest plot indicating per-cohort and meta-analysed associations of pathogen serostatus with brain volume. Model 1 included adjustments for total intracranial volume and other technical covariates; model 2 additionally for age, sex, and ethnicity; and model 3 additionally for BMI, smoking status, educational attainment, socioeconomic position, and alcohol intake. Studies are indicated by colour. Results for Kaposi's sarcoma-associated herpesvirus and pathogen burden indices not shown due to confidence intervals rendering plot uninterpretable, however these data (and all other results) are available in Supplementary Data. Abbreviations: BK=BK virus; CMV=Cytomegalovirus; Ct=*C.trachomatis,* EBV=Epstein-Barr virus; HHV=Human herpesvirus; Hp=*H.pylori;* HPV=Human papillomavirus; HSV=Herpes-simplex virus; JC=John Cunningham virus; MCV=Merkel Cell virus; NSHD=National Survey of Health and Development; SABRE=Southall and Brent Revisited; Tg=*T.gondii;* UKB=UK Biobank; VZV=Varicella zoster virus.

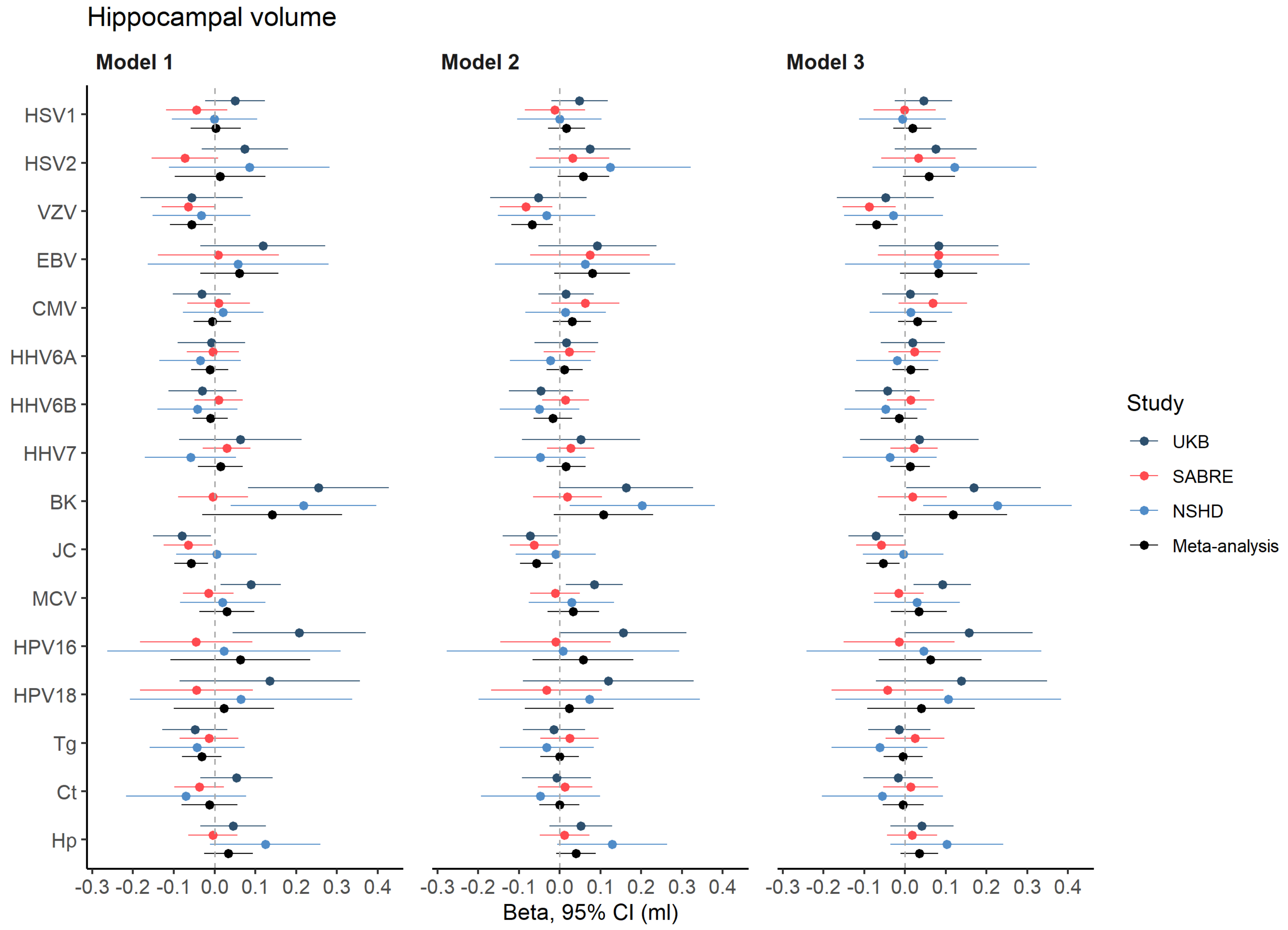

Supplementary Figure 3. Forest plot indicating per-cohort and meta-analysed associations of pathogen serostatus with hippocampal volume. Model 1 included adjustments for total intracranial volume and other technical covariates; model 2 additionally for age, sex, and ethnicity; and model 3 additionally for BMI, smoking status, educational attainment, socioeconomic position, and alcohol intake. Studies are indicated by colour. Results for Kaposi's sarcoma-associated herpesvirus and pathogen burden indices not shown due to confidence intervals rendering plot uninterpretable, however these data (and all other results) are available in Supplementary Data. Abbreviations: BK=BK virus; CMV=Cytomegalovirus; Ct=*C.trachomatis,* EBV=Epstein-Barr virus; HHV=Human herpesvirus; Hp=*H.pylori;* HPV=Human papillomavirus; HSV=Herpes-simplex virus; JC=John Cunningham virus; MCV=Merkel Cell virus; NSHD=National Survey of Health and Development; SABRE=Southall and Brent Revisited; Tg=*T.gondii;* UKB=UK Biobank; VZV=Varicella zoster virus.

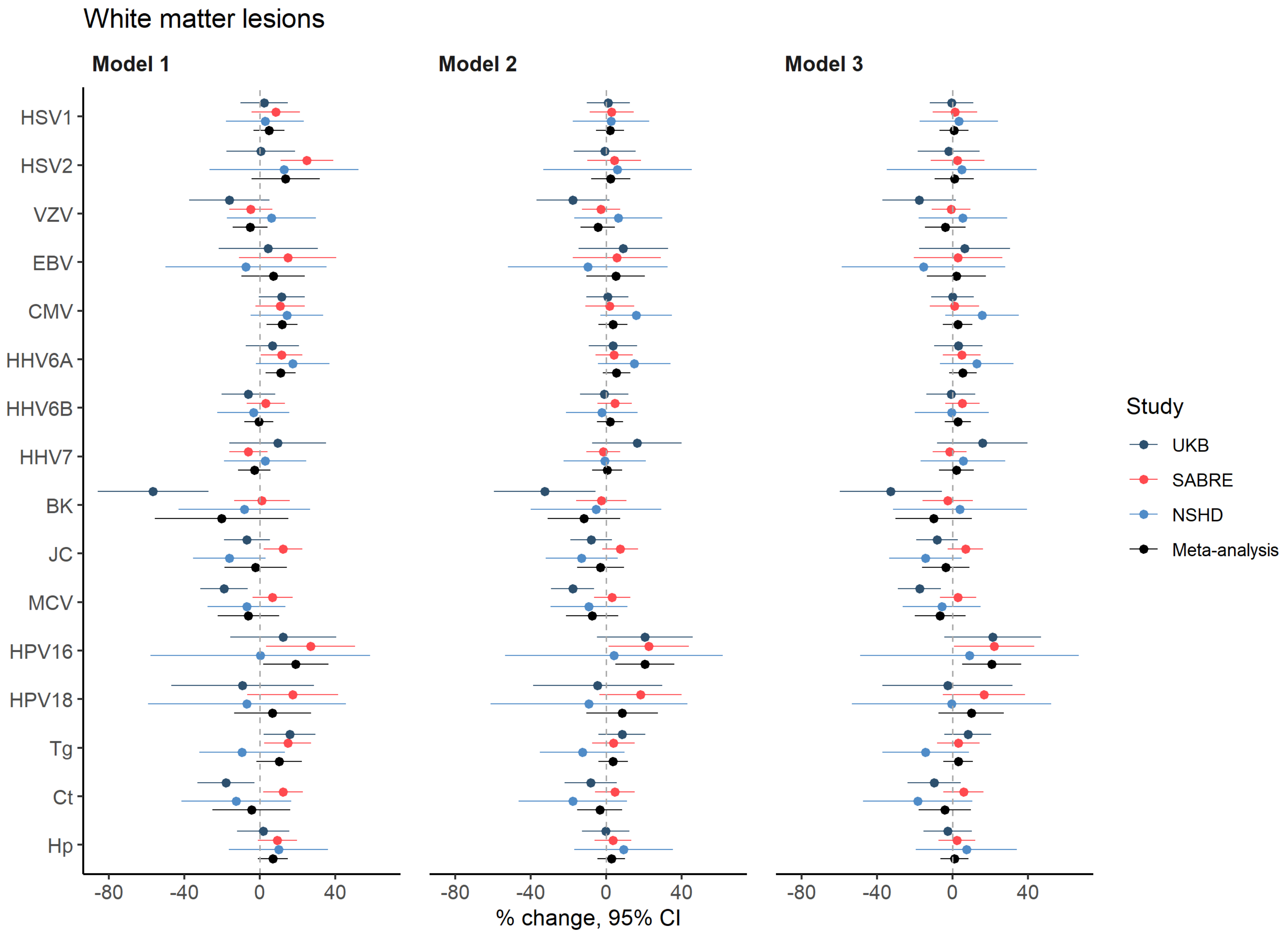

Supplementary Figure 4. Forest plot indicating per-cohort and meta-analysed associations of pathogen serostatus with white matter lesions. Model 1 included adjustments for total intracranial volume and other technical covariates; model 2 additionally for age, sex, and ethnicity; and model 3 additionally for BMI, smoking status, educational attainment, socioeconomic position, and alcohol intake. Studies are indicated by colour. Results for Kaposi's sarcoma-associated herpesvirus and pathogen burden indices not shown due to confidence intervals rendering plot uninterpretable, however these data (and all other results) are available in Supplementary Data. Abbreviations: BK=BK virus; CMV=Cytomegalovirus; Ct=*C.trachomatis,* EBV=Epstein-Barr virus; HHV=Human herpesvirus; Hp=*H.pylori;* HPV=Human papillomavirus; HSV=Herpes-simplex virus; JC=John Cunningham virus; MCV=Merkel Cell virus; NSHD=National Survey of Health and Development; SABRE=Southall and Brent Revisited; Tg=*T.gondii;* UKB=UK Biobank; VZV=Varicella zoster virus.

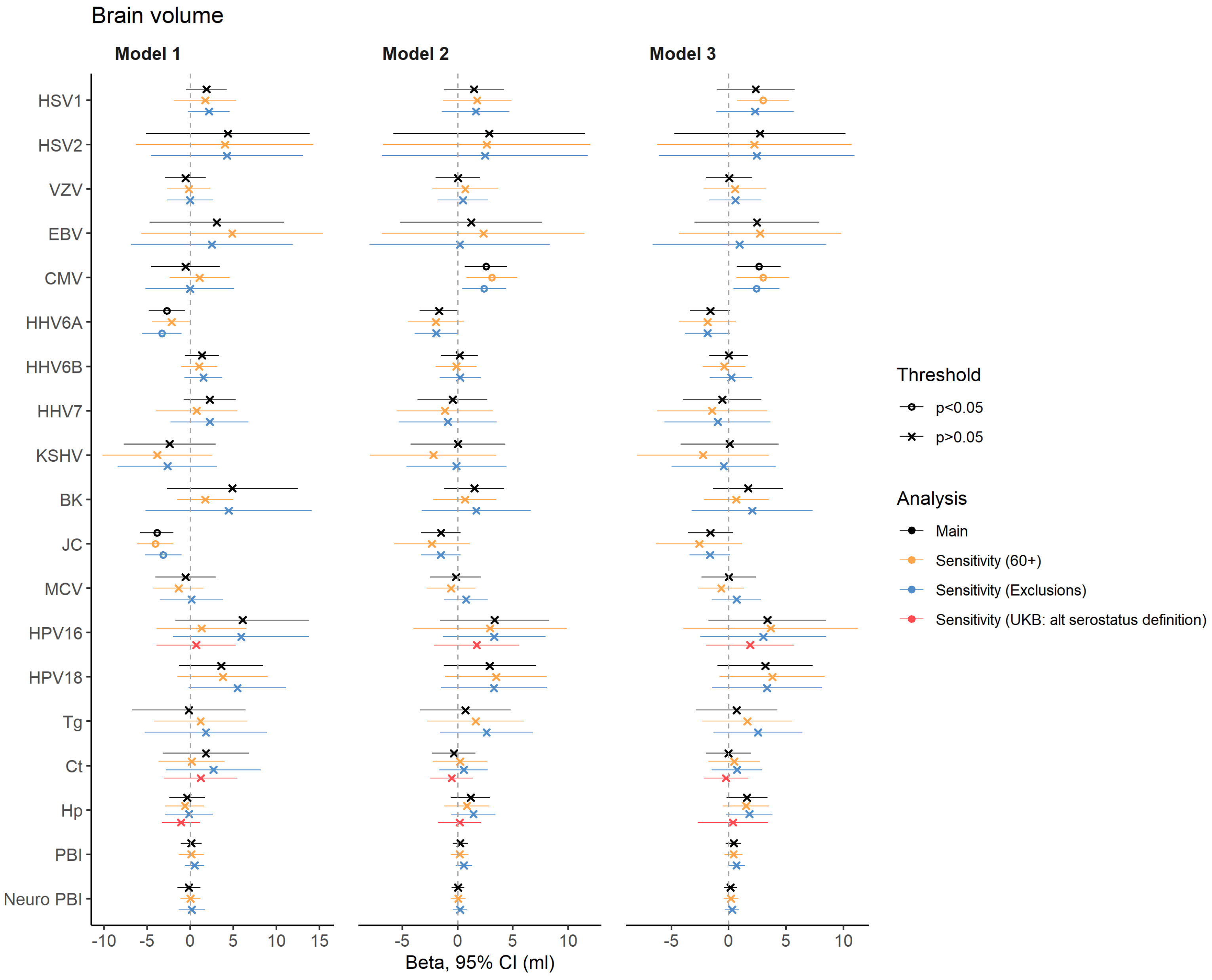

Supplementary Figure 5 (left). Forest plot indicating results from primary and sensitivity analyses of pathogen serostatus with brain volume. Model 1 included adjustments for total intracranial volume and other technical covariates; model 2 additionally for age, sex, and ethnicity; and model 3 additionally for BMI, smoking status, educational attainment, socioeconomic position, and alcohol intake. Analyses are indicated by colour. All results are available in Supplementary Data. Abbreviations: BK=BK virus; CMV=Cytomegalovirus; Ct=*C.trachomatis,* EBV=Epstein-Barr virus; HHV=Human herpesvirus; Hp=*H.pylori;* HPV=Human papillomavirus; HSV=Herpes-simplex virus; JC=John Cunningham virus; KSHV=Kaposi's sarcoma-associated herpesvirus; MCV=Merkel Cell virus; PBI=pathogen burden index; Tg=*T.gondii;* UKB=UK Biobank;VZV=Varicella zoster virus.

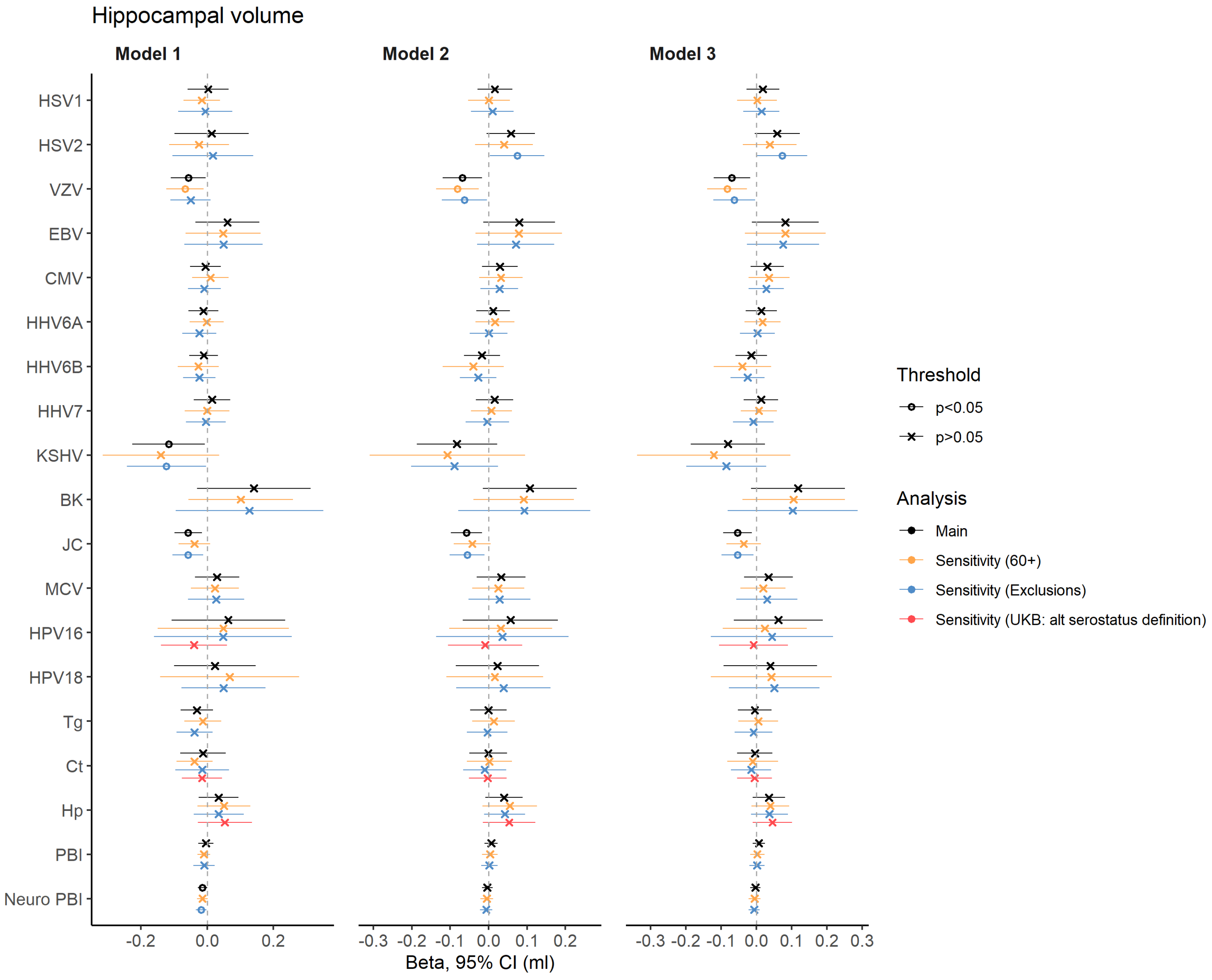

Supplementary Figure 6 (left). Forest plot indicating results from primary and sensitivity analyses of pathogen serostatus with hippocampal volume. Model 1 included adjustments for total intracranial volume and other technical covariates; model 2 additionally for age, sex, and ethnicity; and model 3 additionally for BMI, smoking status, educational attainment, socioeconomic position, and alcohol intake. Analyses are indicated by colour. All results are available in Supplementary Data. Abbreviations: BK=BK virus; CMV=Cytomegalovirus; Ct=*C.trachomatis,* EBV=Epstein-Barr virus; HHV=Human herpesvirus; Hp=*H.pylori;* HPV=Human papillomavirus; HSV=Herpes-simplex virus; JC=John Cunningham virus; KSHV=Kaposi's sarcoma-associated herpesvirus; MCV=Merkel Cell virus; PBI=pathogen burden index; Tg=*T.gondii;* UKB=UK Biobank;VZV=Varicella zoster virus.

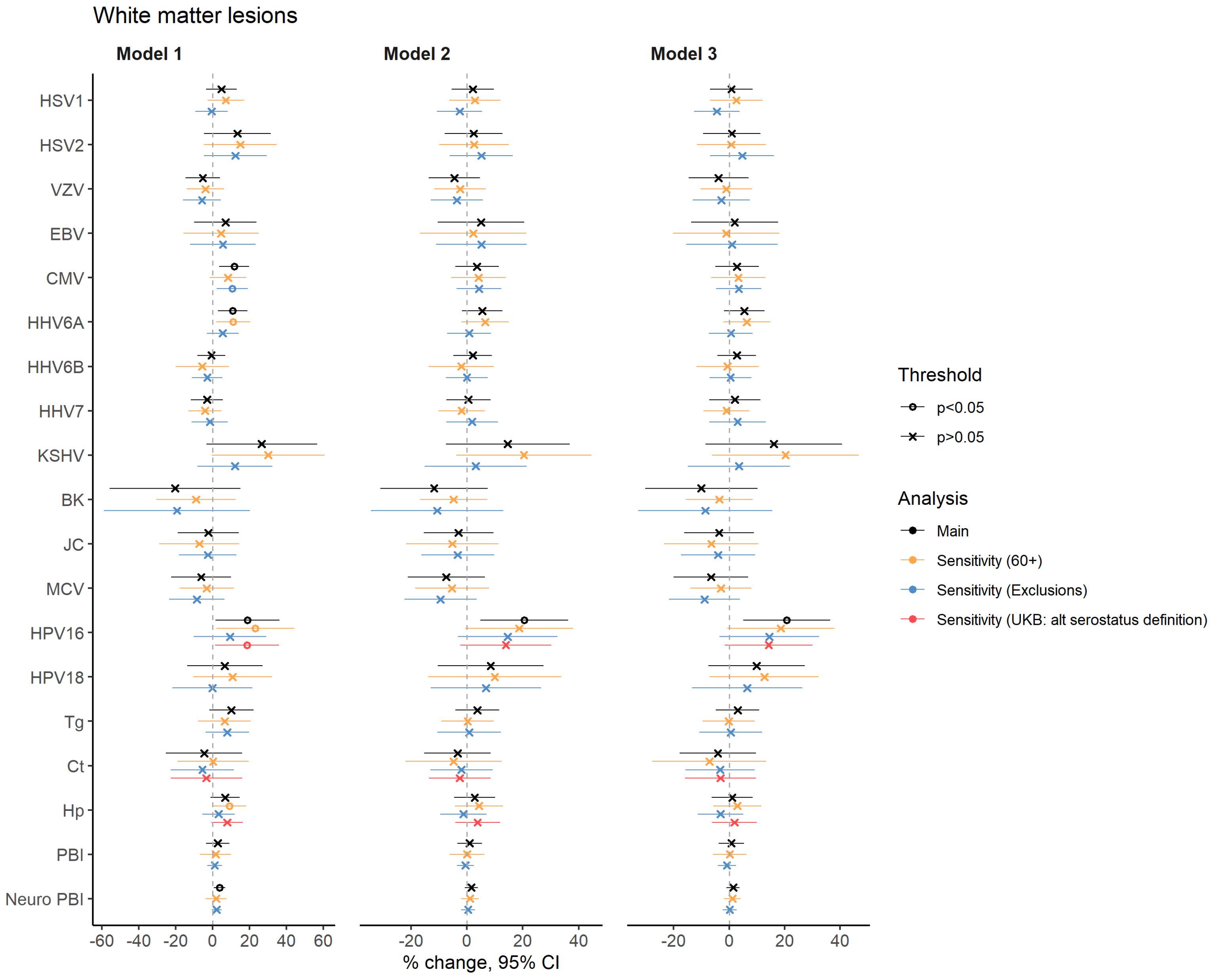

Supplementary Figure 7 (left). Forest plot indicating results from primary and sensitivity analyses of pathogen serostatus with white matter lesions. Model 1 included adjustments for total intracranial volume and other technical covariates; model 2 additionally for age, sex, and ethnicity; and model 3 additionally for BMI, smoking status, educational attainment, socioeconomic position, and alcohol intake. Analyses are indicated by colour. All results are available in Supplementary Data. Abbreviations: BK=BK virus; CMV=Cytomegalovirus; Ct=*C.trachomatis,* EBV=Epstein-Barr virus; HHV=Human herpesvirus; Hp=*H.pylori;* HPV=Human papillomavirus; HSV=Herpes-simplex virus; JC=John Cunningham virus; KSHV=Kaposi's sarcoma-associated herpesvirus; MCV=Merkel Cell virus; PBI=pathogen burden index; Tg=*T.gondii;* UKB=UK Biobank;VZV=Varicella zoster virus.

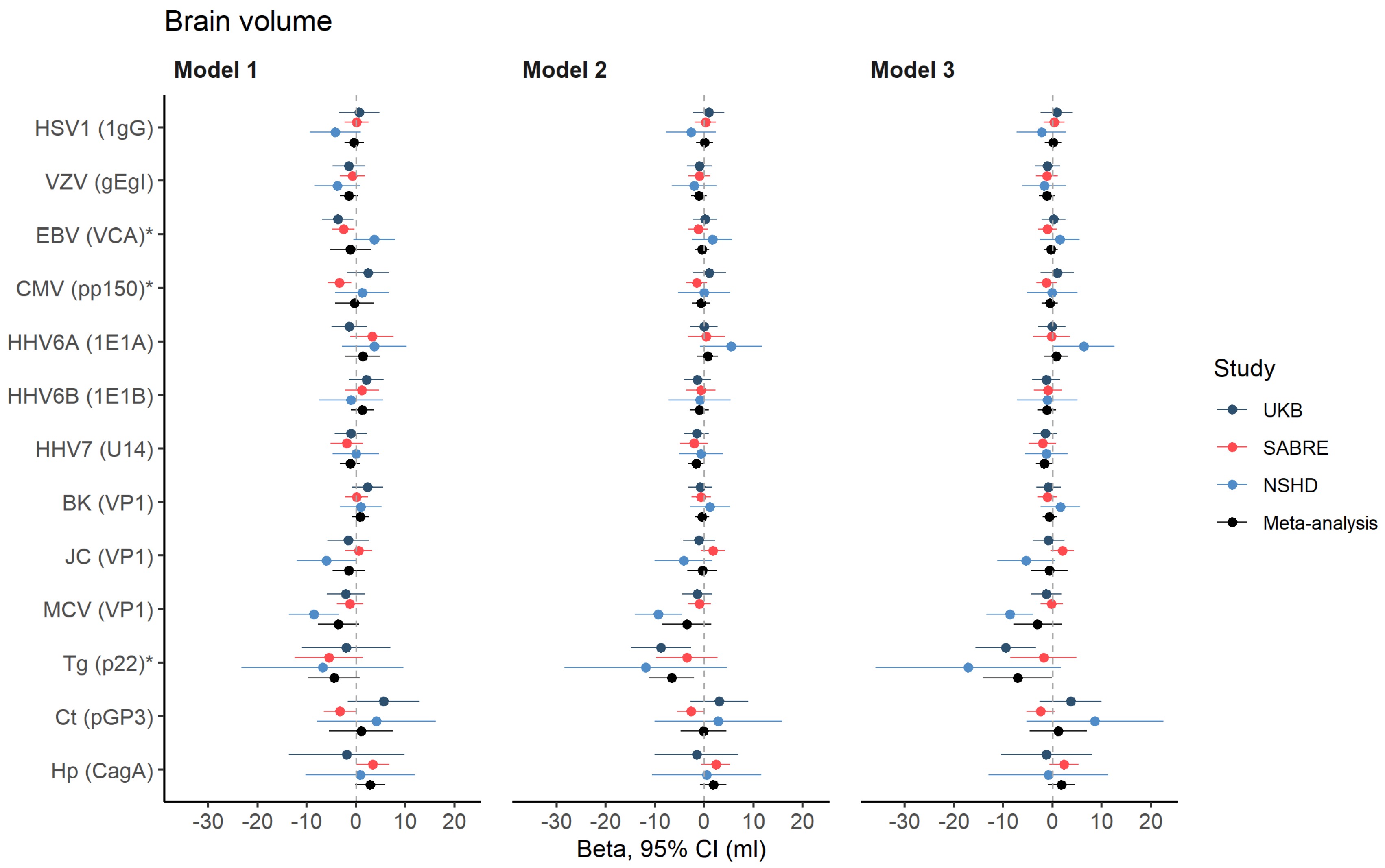

Supplementary Figure 8. Forest plot indicating per-cohort and meta-analysed associations of seroreactivity tertiles with brain volume. Model 1 included adjustments for total intracranial volume and other technical covariates; model 2 additionally for age, sex, and ethnicity; and model 3 additionally for BMI, smoking status, educational attainment, socioeconomic position, and alcohol intake. Pathogens and specific antigens (in brackets) are indicated on the y-axis. Studies are indicated by colour. HSV2 not shown due to large confidence intervals rendering plot uninterpretable, however these data (and all other results) are available as Supplementary Data. Abbreviations: BK=BK virus; CMV=Cytomegalovirus; Ct=*C.trachomatis*, EBV=Epstein-Barr virus; HHV=Human herpesvirus; Hp=*H.pylori;* HPV=Human papillomavirus; HSV=Herpes-simplex virus; JC=John Cunningham virus; MCV=Merkel Cell virus; NSHD=National Survey of Health and Development; SABRE=Southall and Brent Revisited; Tg=*T.gondii*; UKB=UK Biobank; VZV=Varicella zoster virus.

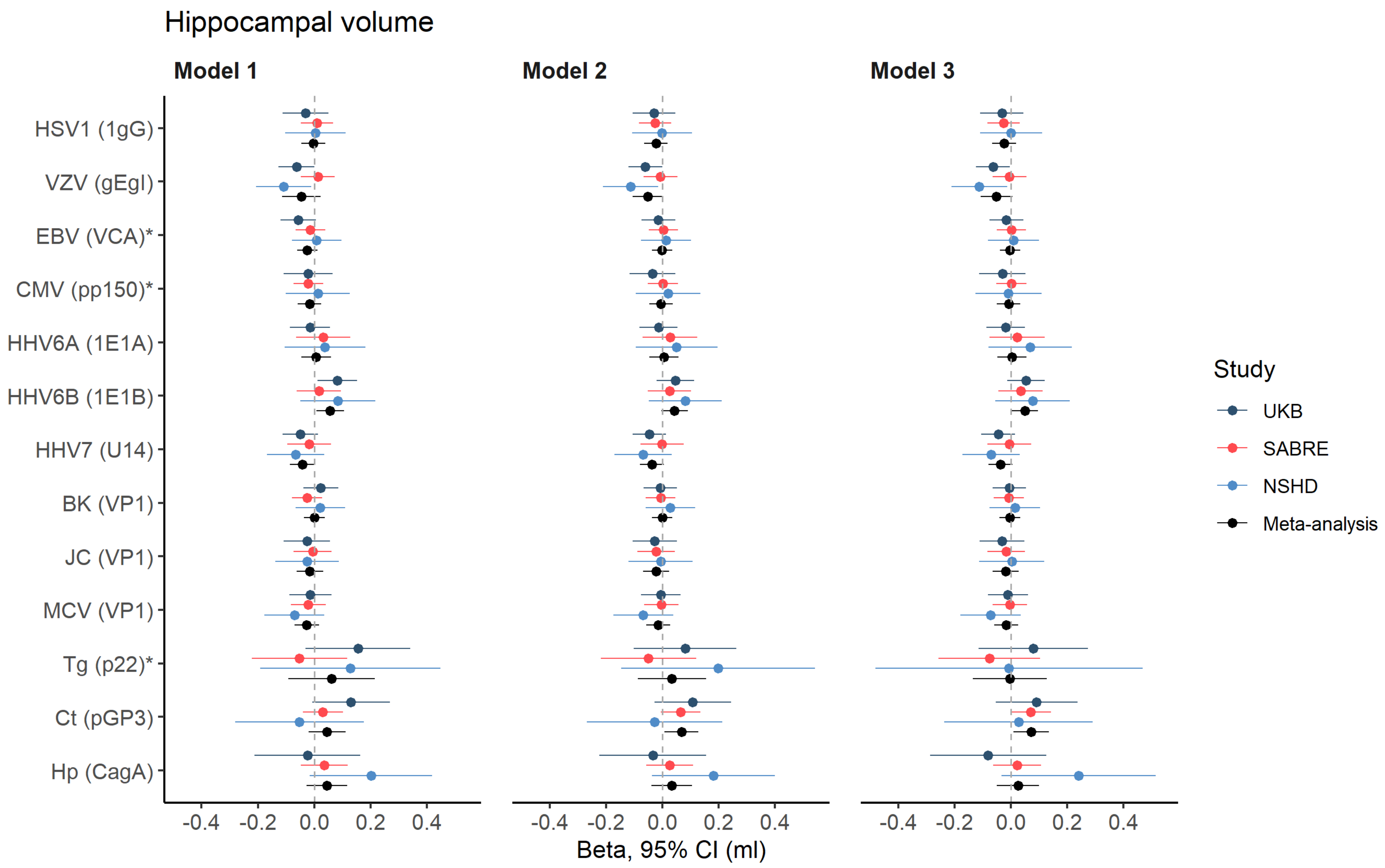

Supplementary Figure 9. Forest plot indicating per-cohort and meta-analysed associations of seroreactivity tertiles with hippocampal volume. Model 1 included adjustments for total intracranial volume and other technical covariates; model 2 additionally for age, sex, and ethnicity; and model 3 additionally for BMI, smoking status, educational attainment, socioeconomic position, and alcohol intake. Pathogens and specific antigens (in brackets) are indicated on the y-axis. Studies are indicated by colour. HSV2 not shown due to large confidence intervals rendering plot uninterpretable, however these data (and all other results) are available as Supplementary Data. Abbreviations: BK=BK virus; CMV=Cytomegalovirus; Ct=*C.trachomatis*, EBV=Epstein-Barr virus; HHV=Human herpesvirus; Hp=*H.pylori;* HPV=Human papillomavirus; HSV=Herpes-simplex virus; JC=John Cunningham virus; MCV=Merkel Cell virus; NSHD=National Survey of Health and Development; SABRE=Southall and Brent Revisited; Tg=*T.gondii*; UKB=UK Biobank; VZV=Varicella zoster virus.

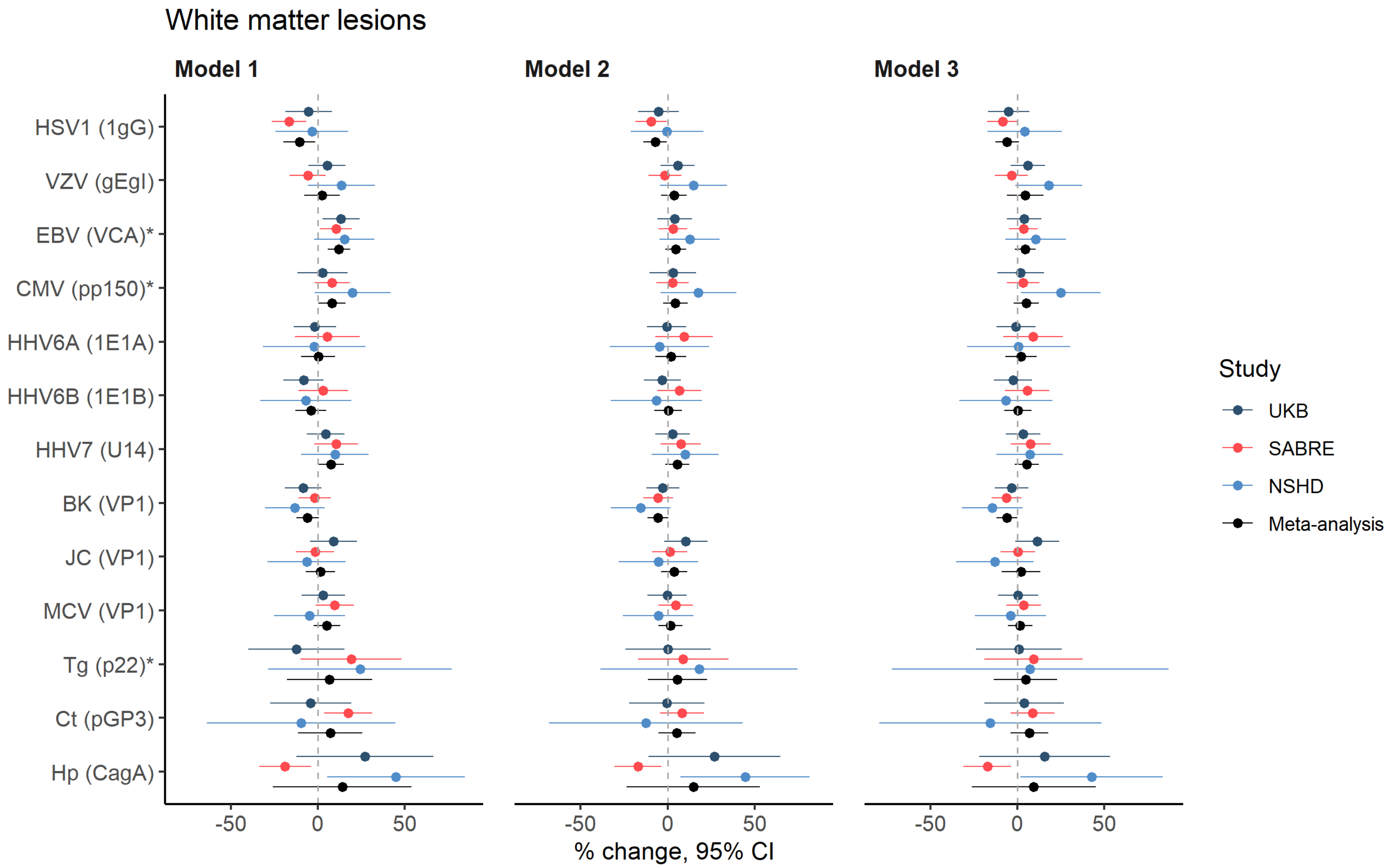

Supplementary Figure 10. Forest plot indicating per-cohort and meta-analysed associations of seroreactivity tertiles with white matter lesions. Model 1 included adjustments for total intracranial volume and other technical covariates; model 2 additionally for age, sex, and ethnicity; and model 3 additionally for BMI, smoking status, educational attainment, socioeconomic position, and alcohol intake. Pathogens and specific antigens (in brackets) are indicated on the y-axis. Studies are indicated by colour. HSV2 not shown due to large confidence intervals rendering plot uninterpretable, however these data (and all other results) are available as Supplementary Data. Abbreviations: BK=BK virus; CMV=Cytomegalovirus; Ct=*C.trachomatis*, EBV=Epstein-Barr virus; HHV=Human herpesvirus; Hp=*H.pylori;* HPV=Human papillomavirus; HSV=Herpes-simplex virus; JC=John Cunningham virus; MCV=Merkel Cell virus; NSHD=National Survey of Health and Development; SABRE=Southall and Brent Revisited; Tg=*T.gondii*; UKB=UK Biobank; VZV=Varicella zoster virus.

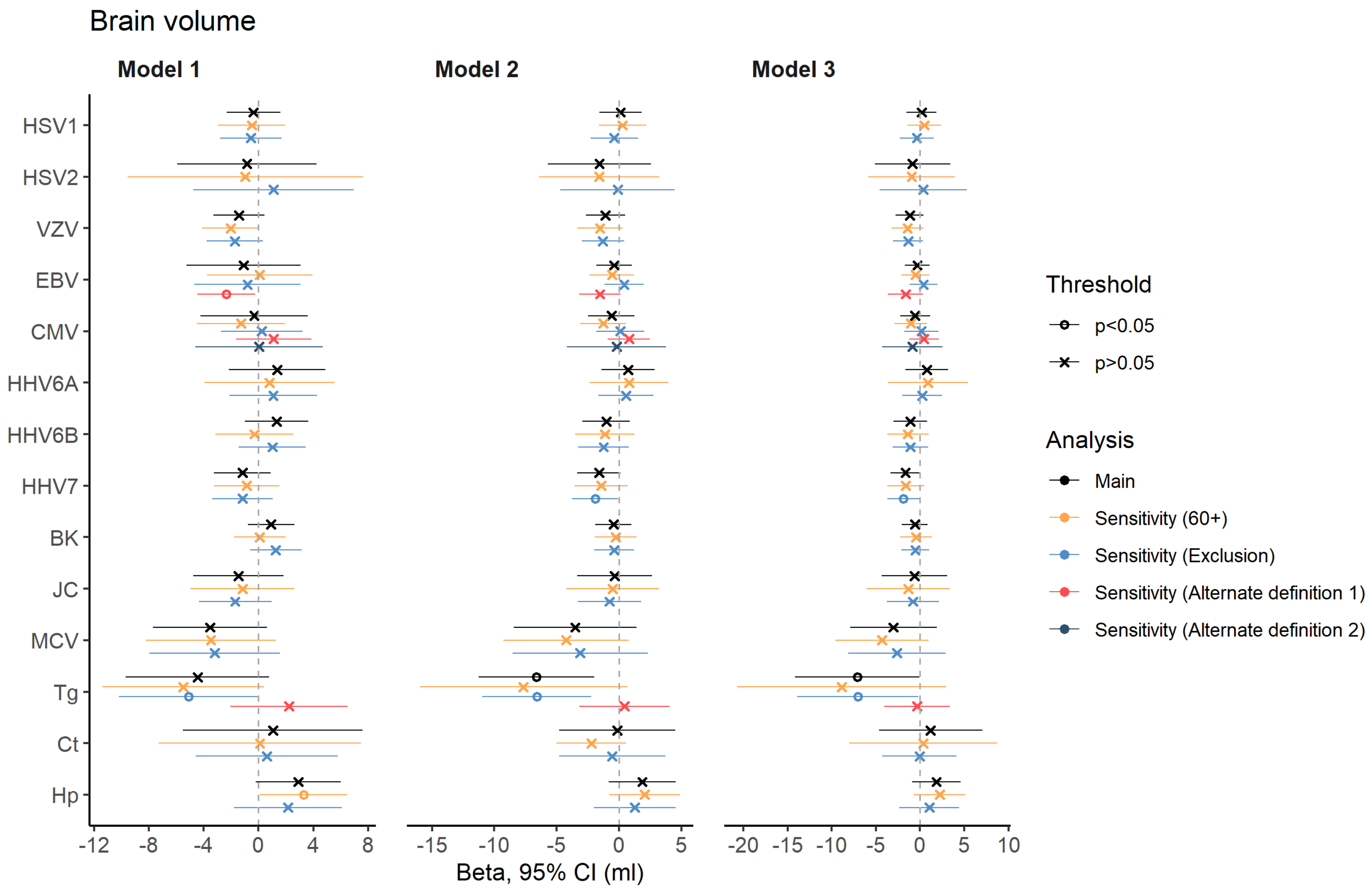

Supplementary Figure 11. Forest plot indicating results from primary and sensitivity analyses of pathogen seroreactivity with brain volume. Model 1 included adjustments for total intracranial volume and other technical covariates; model 2 additionally for age, sex, and ethnicity; and model 3 additionally for BMI, smoking status, educational attainment, socioeconomic position, and alcohol intake. Analyses are indicated by colour. Model 3 (sensitivity 60+) would not run in UK Biobank for Ct (model 3) due to insufficient degrees of freedom. All results are available in Supplementary Data. Abbreviations: BK=BK virus; CMV=Cytomegalovirus; Ct=*C.trachomatis*, EBV=Epstein-Barr virus; HHV=Human herpesvirus; Hp=*H.pylori*; HPV=Human papillomavirus; HSV=Herpes-simplex virus; JC=John Cunningham virus; MCV=Merkel Cell virus; Tg=*T.gondii*; UKB=UK Biobank; VZV=Varicella zoster virus.

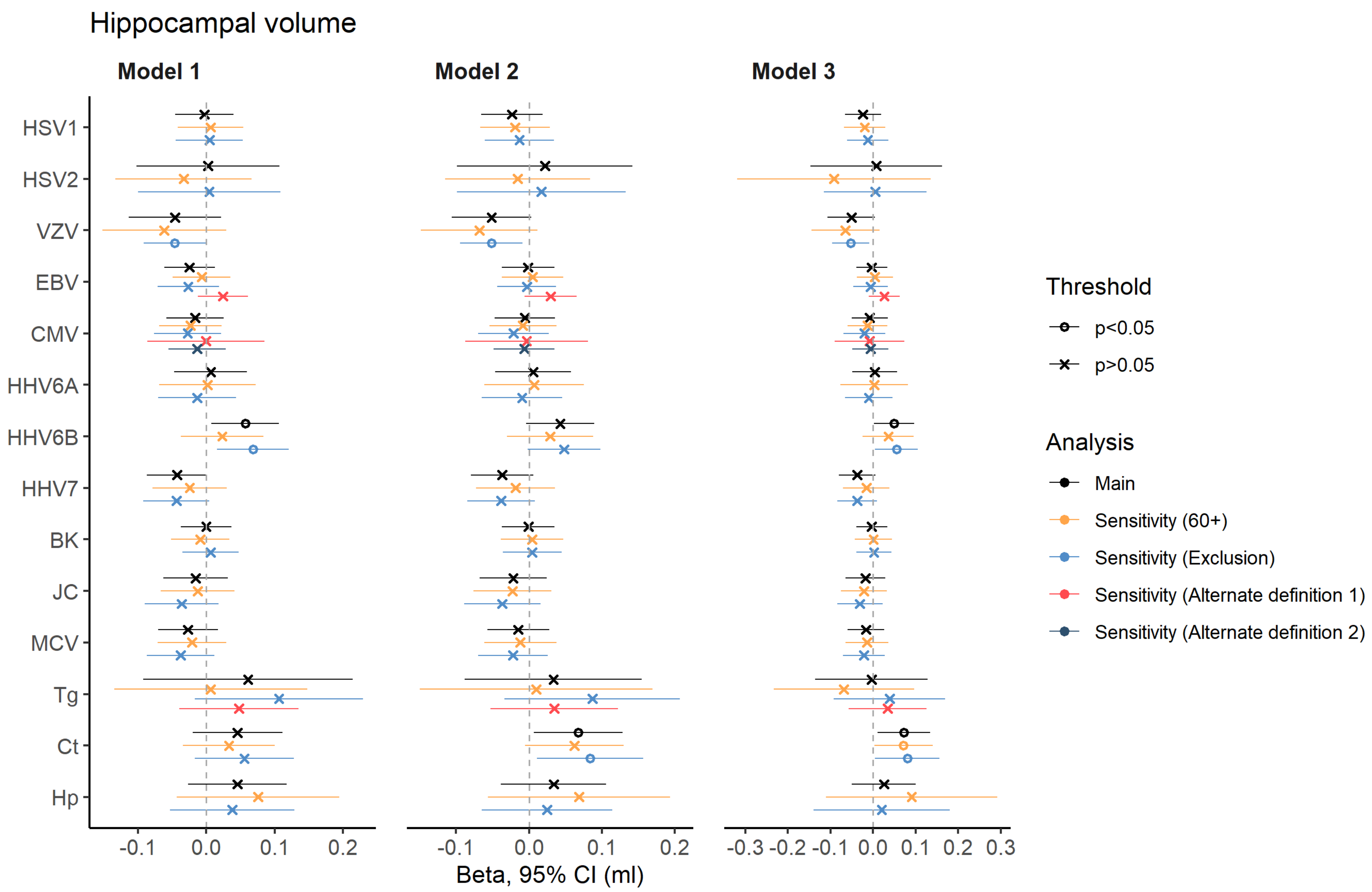

Supplementary Figure 12. Forest plot indicating results from primary and sensitivity analyses of pathogen seroreactivity with hippocampal volume. Model 1 included adjustments for total intracranial volume and other technical covariates; model 2 additionally for age, sex, and ethnicity; and model 3 additionally for BMI, smoking status, educational attainment, socioeconomic position, and alcohol intake. Analyses are indicated by colour. Model 3 (sensitivity 60+) would not run in UK Biobank for Ct (model 3) due to insufficient degrees of freedom. All results are available in Supplementary Data. Abbreviations: BK=BK virus; CMV=Cytomegalovirus; Ct=*C.trachomatis*, EBV=Epstein-Barr virus; HHV=Human herpesvirus; Hp=*H.pylori*; HPV=Human papillomavirus; HSV=Herpes-simplex virus; JC=John Cunningham virus; MCV=Merkel Cell virus; Tg=*T.gondii*; UKB=UK Biobank; VZV=Varicella zoster virus.

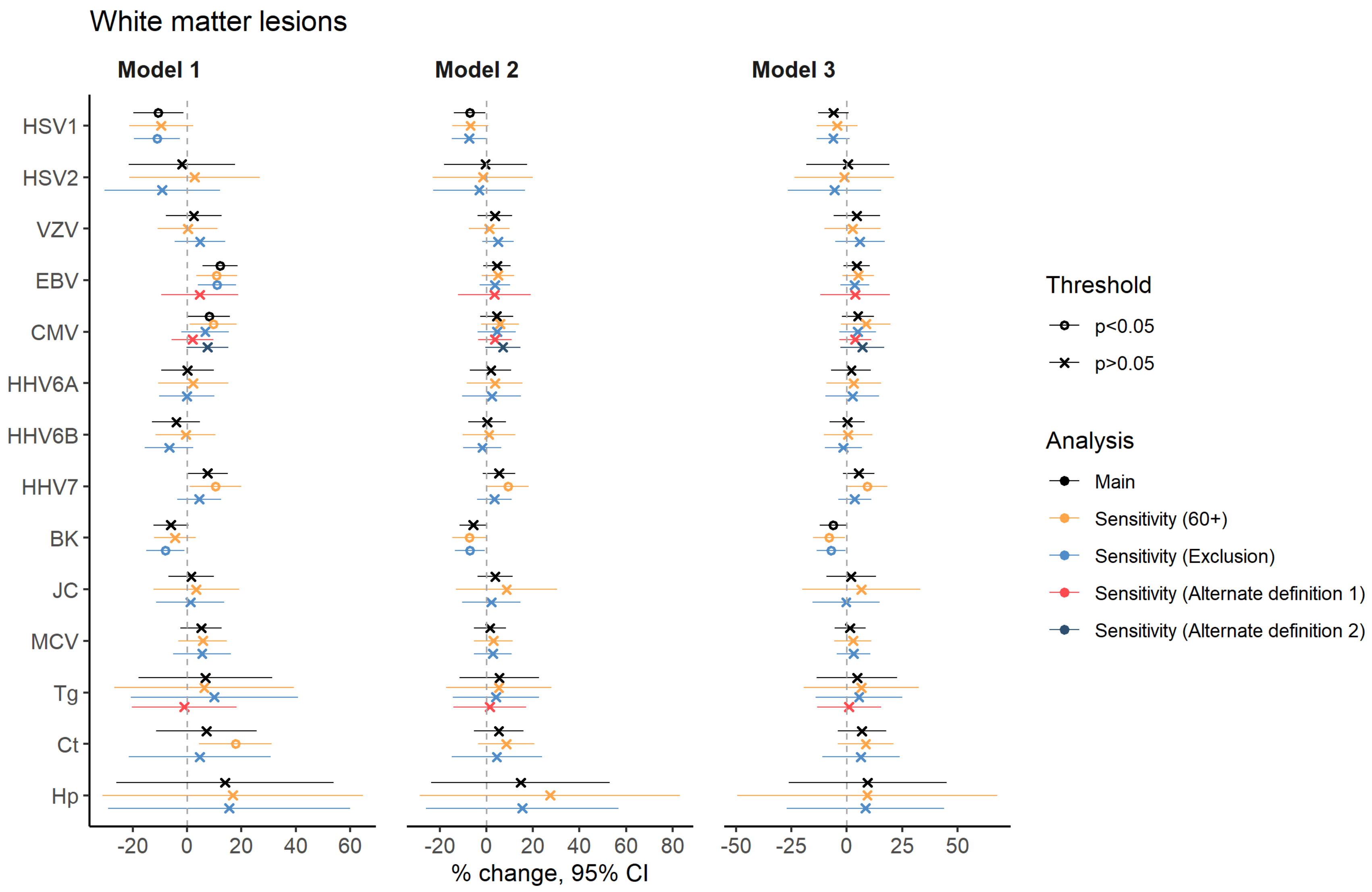

Supplementary Figure 13. Forest plot indicating results from primary and sensitivity analyses of pathogen seroreactivity with white matter lesions. Model 1 included adjustments for total intracranial volume and other technical covariates; model 2 additionally for age, sex, and ethnicity; and model 3 additionally for BMI, smoking status, educational attainment, socioeconomic position, and alcohol intake. Analyses are indicated by colour. Model 3 (sensitivity 60+) would not run in UK Biobank for Ct (model 3) due to insufficient degrees of freedom. All results are available in Supplementary Data. Abbreviations: BK=BK virus; CMV=Cytomegalovirus; Ct=*C.trachomatis*, EBV=Epstein-Barr virus; HHV=Human herpesvirus; Hp=*H.pylori*; HPV=Human papillomavirus; HSV=Herpes-simplex virus; JC=John Cunningham virus; MCV=Merkel Cell virus; Tg=*T.gondii*; UKB=UK Biobank; VZV=Varicella zoster virus.
