# Supplementary material for: Common infections and neuroimaging markers of dementia in three UK cohort studies
Source: Alzheimers Dement. 2024 Jan 22;20(3):2128–42. doi: 10.1002/alz.13613 (PMC10984486; doi:10.1002/alz.13613)
Supplement: Supplementary file 3 — Supporting Information [file ALZ-20-2128-s001.pdf]

Supplementary Tables

Supplementary Table 1. Characteristics of participants and seroprevalence for the 17 pathogens common to all cohorts.

		**NSHD**		**SABRE**		**UKB**	
		Serology	*Serology & Imaging*	*Serology*	*Serology & Imaging*	*Serology*	*Serology & Imaging*
**N**		1793	438	1410	1259	9689	935
**Sex (% female)**		50.5	47.9	23.7	22.8	55.9	55.9
**Age at serology in years, mean (SD)**		63.2 (1.1)	63.3 (1.1)	69.6 (6.1)	69.6 (6.1)	56.7 (8.2)	55.3 (7.7)
**Herpesviruses**	Herpes simplex virus-1† (% seropositive)	67.3	64.4	81.6	81.1	69.8	61.5
	Herpes simplex virus-2† (% seropositive)	7.3	6.4	15.1	15.7	16.1	12.7
	Varicella zoster virus† (% seropositive)	79.4	78.3	73.7	73	92.4	91.4
	Epstein-Barr virus† (% seropositive)	92.9	94.1	95.8	95.9	94.7	94.4
	Cytomegalovirus† (% seropositive)	54.2	51.8	79.9	80.1	58.2	52.4
	Human herpesvirus-6A† (% seropositive)	42.6	42.5	29.9	29.5	77.5	76.1
	Human herpesvirus-6B† (% seropositive)	53.9	50.9	43.7	44.2	79.2	77.1
	Human betaherpesvirus-7† (% seropositive)	72.7	74.0	47.2	46.3	94.7	94.2
	Kaposi's sarcoma-	Low cell count	Low cell count	2.0	1.9	8.1	8.0

	associated virus† (% seropositive)						
**Bacteria/Protozoa**	*C.trachomatis* (% seropositive)	17.9	13.5	41.8	42.6	21.4	19.7
	H.pylori (% seropositive)	17.6	14.6	39.5	39.2	31.5	25.8
	T.gondii† (% seropositive)	24.4	23.5	21.2	20.8	28.0	25.9
**Human Polyomaviruses**	BK virus (% seropositive)	91.4	92.2	86.5	86.5	95.4	95.7
	JC virus† (% seropositive)	51.6	54.3	59.7	60.4	57.4	57.2
	Merkel Cell virus (% seropositive)	59.7	65.5	66.6	66.6	66.6	65.2
**Human Papillomaviruses**	Human Papillomavirus-16 (% seropositive)	2.7	2.7	4.8	4.8	4.4	4.8
	Human Papillomavirus-18 (% seropositive)	2.4	3.4	4.6	4.7	2.7	2.6
**Pathogen burden index (mean (SD))**		0.43 (0.12)	0.43 (0.12)	0.47 (0.13)	0.47 (0.13)	0.53 (0.12)	0.51 (0.12)
**Neurotropic pathogen burden index mean (SD))**		0.50 (0.15)	0.49 (0.15)	0.50 (0.14)	0.50 (0.14)	0.61 (0.15)	0.59 (0.15)
**Ethnicity, N (%)**		White*	White*	African Caribbean: 15.9 European: 47.8 South Asian: 36.3 Missing N: 4	African Caribbean: 16.2 European: 46.7 South Asian: 37.1	Asian or Asian British: 2.1 Black or Black British: 1.5 Chinese: 0.5	Asian or Asian British: 1.4 Black or Black British: 0.7 Chinese: 0.4

Supplementary Tables

						Mixed: 0.5<br>Other: 0.8<br>White: 94.7<br>Missing N: 45	Mixed: 0.4<br>Other: 0.4<br>White: 96.6<br>Missing N: 1
**Body mass index in kg/m^2, mean (SD)**		27.7 (4.7)<br>Missing N: 5	27.5 (4.1)	27.5 (4.7)<br>Missing N: 7	27.4 (4.5)	27.3 (4.8)<br>Missing N: 32	26.5 (4.2)
**Alcohol intake, N (%)**	Low	54.9	50.2	70.9	70.6	52	48.1
	Moderate	24.3	26.7	15.2	15.3	24.1	27
	Heavy	20.7<br>Missing N: 55	23<br>Missing N: 4	13.9<br>Missing N: 44	14.1<br>Missing N: 33	23.9<br>Missing N: 139	24.9<br>Missing N: 5
**Ever smoker, N (%)**		53<br>Missing M: 42	45.9<br>Missing N: 7	43.3<br>Missing N: 14	42.6<br>Missing N: 8	44.2<br>Missing N: 54	37.5<br>Missing N: 2
**Highest educational attainment, N (%)**	None	36.2	20.4	31.1	30.1	17.2	7.3
	Up to ordinary ('O') level, or equivalent	20.8	24.6	20.1	20.1	27	21.6
	Advanced ('A') level or equivalent, or higher	43<br>Missing N: 89	54.9<br>Missing N: 12	48.8<br>Missing N: 239	49.9<br>Missing N: 212	55.8<br>Missing N: 105	71.1<br>Missing N: 1
**Socioeconomic position, N (%)**	Professional	7.9	12.6	3.5	3.7	Townsend quintiles<br>Missing N: 8	Townsend quintiles<br>Missing N: 1
	Intermediate	42	51.8	20.5	20.7		
	Nonmanual skilled	23	20.8	15	14		
	Manual skilled	14.6	9.1	35.3	35.3		

Supplementary Tables

	Partly skilled or Unskilled‡	12.4<br>Missing N: 11	5.7	25.4<br>Missing N: 19	25.3<br>Missing N: 14		

Abbreviations: NSHD = National Survey of Health and Development; SABRE = Southall and Brent Revisited; UKB = UK Biobank. *The NSHD is predominantly white British as they are representative of births in mainland Britain in 1946, which was prior to major immigration flows [13]. † Pathogen considered to be neurotropic. ‡Categories collapsed due to low cell counts for the purpose of this table only

Supplementary Tables

Supplementary Table 2. Meta-analysed associations of serostatus and pathogen burden scores with brain volume.
Estimates, 95% confidence intervals, p values, and I^2 statistics are presented. For pathogen burden analyses, estimates represent the difference in outcome per 0.1 unit increase in pathogen burden (proportion of pathogens or neurotropic pathogens seropositive to). Associations significant at p<0.05 are in bold, and those significant following multiple testing correction are further indicated by an asterisk. Model 1 included adjustments for total intracranial volume and other technical covariates; model 2 additionally for age, sex, and ethnicity; and model 3 additionally for BMI, smoking status, educational attainment, socioeconomic position, and alcohol intake.

	**Brain volume**								
	**Model 1**			**Model 2**			**Model 3**		
	β [95% CI] (ml)	p	I^2	β [95% CI (ml)	p	I^2	β [95% CI] (ml)	p	I^2
**HSV1**	1.87 [-0.49,4.22]	0.12	11	1.48 [-1.26,4.23]	0.29	50	2.35 [-1.04,5.73]	0.17	67
**HSV2**	4.33 [-5.18,13.85]	0.37	88	2.87 [-5.81,11.54]	0.52	89	2.73 [-4.73,10.19]	0.47	85
**VZV**	-0.58 [-2.95,1.79]	0.63	0	0.03 [-2,2.07]	0.97	0	0.04 [-1.97,2.06]	0.97	0
**EBV**	3.07 [-4.74,10.88]	0.44	64	1.21 [-5.19,7.62]	0.71	61	2.45 [-2.98,7.88]	0.38	46
**CMV**	-0.56 [-4.56,3.43]	0.78	69	**2.57 [0.66,4.48]**	**0.008**	0	**2.62 [0.71,4.53]**	**0.007**	0
**HHV6A**	**-2.74 [-4.82,-0.66]**	**0.01**	0	-1.68 [-3.45,0.09]	0.06	0	-1.61 [-3.37,0.15]	0.07	0
**HHV6B**	1.36 [-0.63,3.34]	0.18	0	0.17 [-1.52,1.85]	0.85	0	0 [-1.68,1.67]	1	0
**HHV7**	2.24 [-0.79,5.26]	0.15	26	-0.46 [-3.62,2.71]	0.78	41	-0.56 [-3.99,2.86]	0.75	49

Supplementary Tables

**KSHV**	-2.4 [-7.75,2.94]	0.38	0	0.02 [-4.26,4.31]	0.99	0	0.07 [-4.21,4.35]	0.97	0
**HPV16**	6.05 [-1.71,13.81]	0.13	57	3.35 [-1.6,8.29]	0.18	30	3.37 [-1.76,8.51]	0.2	35
**HPV18**	3.58 [-1.31,8.46]	0.15	0	2.9 [-1.27,7.07]	0.17	0	3.18 [-0.97,7.32]	0.13	0
**BK**	4.87 [-2.71,12.45]	0.21	72	1.5 [-1.22,4.23]	0.28	0	1.69 [-1.37,4.74]	0.28	10
**JC**	**-3.89 [-5.81,-1.97]**	**7E-05***	0	-1.51 [-3.31,0.29]	0.1	12	-1.59 [-3.55,0.36]	0.11	24
**MCV**	-0.57 [-4.07,2.94]	0.75	62	-0.16 [-2.47,2.14]	0.89	39	0 [-2.38,2.37]	1	43
**Ct**	1.8 [-3.2,6.8]	0.48	73	-0.35 [-2.32,1.61]	0.73	0	-0.03 [-1.98,1.93]	0.98	0
**Tg**	-0.18 [-6.77,6.42]	0.96	87	0.69 [-3.42,4.8]	0.74	75	0.67 [-2.88,4.22]	0.71	67
**Hp**	-0.36 [-2.45,1.72]	0.73	0	1.18 [-0.61,2.96]	0.2	0	1.59 [-0.2,3.38]	0.08	0
**PBI**	0.12 [-1.1,1.34]	0.85	53	0.24 [-0.45,0.92]	0.5	0	0.42 [-0.26,1.11]	0.22	0
**Neuro PBI**	-0.18 [-1.51,1.16]	0.8	73	0.03 [-0.54,0.6]	0.91	0	0.15 [-0.42,0.72]	0.6	0

Abbreviations: BK=BK virus; CMV=Cytomegalovirus; Ct=*C.trachomatis*; EBV=Epstein-Barr virus; HHV=Human herpesvirus; Hp=*H.pylori*; HPV=Human Papillomavirus; HSV=Herpes simplex virus; JC=John Cunningham virus; KSHV=Kaposi's sarcoma-associated herpesviruses; MCV=Merkel Cell virus; PBI=Pathogen burden index; Tg=*T.gondii*; VZV=Varicella zoster virus. The neuro PBI included the nine herpesviruses, JC virus, and *T.gondii*.

Supplementary Tables

Supplementary Table 3. Meta-analysed associations of serostatus and pathogen burden scores with hippocampal volume.
Estimates, 95% confidence intervals, p values, and I^2 statistics are presented. For pathogen burden analyses, estimates represent the difference in outcome per 0.1 unit increase in pathogen burden (proportion of pathogens or neurotropic pathogens seropositive to). Associations significant at p<0.05 are in bold, and those significant following multiple testing correction are further indicated by an asterisk. Model 1 included adjustments for total intracranial volume and other technical covariates; model 2 additionally for age, sex, and ethnicity; and model 3 additionally for BMI, smoking status, educational attainment, socioeconomic position, and alcohol intake.

	Hippocampal volume								
	Model 1			Model 2			Model 3		
	β [95% CI] (ml)	p	I^2	β [95% CI] (ml)	p	I^2	β [95% CI] (ml)	p	I^2
**HSV1**	0 [-0.06,0.06]	0.93	39	0.02 [-0.03,0.06]	0.49	0	0.02 [-0.03,0.06]	0.44	0
**HSV2**	0.01 [-0.1,0.13]	0.81	63	0.06 [-0.01,0.12]	0.07	0	0.06 [0,0.12]	0.07	0
**VZV**	**-0.06 [-0.11,0]**	**0.03**	0	**-0.07 [-0.12,-0.02]**	**0.009**	0	**-0.07 [-0.12,-0.02]**	**0.008**	0
**EBV**	0.06 [-0.03,0.16]	0.21	0	0.08 [-0.01,0.17]	0.09	0	0.08 [-0.01,0.18]	0.09	0
**CMV**	-0.01 [-0.05,0.04]	0.83	0	0.03 [-0.02,0.08]	0.21	0	0.03 [-0.02,0.08]	0.2	0
**HHV6A**	-0.01 [-0.06,0.03]	0.61	0	0.01 [-0.03,0.06]	0.6	0	0.01 [-0.03,0.06]	0.54	0
**HHV6B**	-0.01 [-0.05,0.03]	0.63	0	-0.02 [-0.06,0.03]	0.47	17	-0.01 [-0.06,0.03]	0.52	8
**HHV7**	0.01 [-0.04,0.07]	0.61	11	0.02 [-0.03,0.06]	0.53	0	0.01 [-0.04,0.06]	0.61	0

Supplementary Tables

**KSHV**	**-0.12 [-0.23,-0.01]**	**0.04**	0	-0.08 [-0.19,0.02]	0.12	0	-0.08 [-0.19,0.02]	0.13	0
**HPV16**	0.06 [-0.11,0.23]	0.47	61	0.06 [-0.07,0.18]	0.37	33	0.06 [-0.06,0.19]	0.33	34
**HPV18**	0.02 [-0.1,0.15]	0.71	17	0.02 [-0.09,0.13]	0.68	4	0.04 [-0.09,0.17]	0.56	27
**BK**	0.14 [-0.03,0.31]	0.11	77	0.11 [-0.01,0.23]	0.08	57	0.12 [-0.01,0.25]†	0.08	62
**JC**	**-0.06 [-0.1,-0.02]**	**0.007**	0	**-0.06 [-0.1,-0.02]**	**0.006**	0	**-0.05 [-0.09,-0.01]**	**0.01**	0
**MCV**	0.03 [-0.04,0.1]	0.38	55	0.03 [-0.03,0.1]	0.31	52	0.03 [-0.04,0.1]†	0.33	59
**Ct**	-0.01 [-0.08,,0.06]	0.73	41	0 [-0.05,0.05]	0.97	0	0 [-0.05,0.05]	0.86	0
**Tg**	-0.03 [-0.08,0.02]	0.2	0	0 [-0.05,0.05]	0.99	0	0 [-0.05,0.04]	0.84	0
**Hp**	0.03 [-0.03,0.09]	0.26	33	0.04 [-0.01,0.09]	0.1	10	0.03 [-0.01,0.08]	0.13	0
**PBI**	0 [-0.03,0.02]	0.71	41	0.01 [-0.01,0.02]	0.43	0	0.01 [-0.01,0.02]	0.44	0
**Neuro PBI**	**-0.01 [-0.03,0]**	**0.05**	0	0 [-0.02,0.01]	0.65	0	0 [-0.02,0.01]	0.66	0

Abbreviations: BK=BK virus; CMV=Cytomegalovirus; Ct=*C.trachomatis*; EBV=Epstein-Barr virus; HHV=Human herpesvirus; Hp=*H.pylori*; HPV=Human Papillomavirus; HSV=Herpes simplex virus; JC=John Cunningham virus; KSHV=Kaposi's sarcoma-associated herpesviruses; MCV=Merkel Cell virus; PBI=Pathogen burden index; Tg=*T.gondii*; VZV=Varicella zoster virus. The neuro PBI included the nine herpesviruses, JC virus, and *T.gondii*.

Supplementary Tables

Supplementary Table 4. Meta-analysed associations of serostatus and pathogen burden scores with white matter lesion volume.
Estimates, 95% confidence intervals, p values, and I^2 statistics are presented. For pathogen burden analyses, estimates represent the difference in outcome per 0.1 unit increase in pathogen burden (proportion of pathogens or neurotropic pathogens seropositive to). Associations significant at $p<0.05$ are in bold, and those significant following multiple testing correction are further indicated by an asterisk. Model 1 included adjustments for total intracranial volume and other technical covariates; model 2 additionally for age, sex, and ethnicity; and model 3 additionally for BMI, smoking status, educational attainment, socioeconomic position, and alcohol intake.

	White matter lesion volume								
	Model 1			Model 2			Model 3		
	% change [95% CI]	p	I^2	% change [95% CI]	p	I^2	% change [95% CI]	p	I^2
**HSV1**	4.85 [-3.39,13.09]	0.25	0	2.08 [-5.47,9.62]	0.59	0	0.8 [-6.88,8.47]	0.84	0
**HSV2**	13.59 [-4.51,31.68]	0.14	55	2.36 [-8.02,12.74]	0.66	0	0.94 [-9.47,11.35]	0.86	0
**VZV**	-5.26 [-14.52,3.99]	0.26	0	-4.47 [-13.63,4.7]	0.34	8	-3.82 [-14.6,6.96]	0.49	25
**EBV**	7.03 [-9.89,23.94]	0.42	0	5.03 [-10.45,20.51]	0.52	0	2 [-13.66,17.66]	0.8	0
**CMV**	11.77 [3.67,19.87]	**0.004**	0	3.61 [-4.15,11.36]	0.36	0	2.84 [-4.98,10.67]	0.48	0
**HHV6A**	10.97 [3.03,18.92]	**0.007**	0	5.5 [-1.77,12.77]	0.14	0	5.48 [-1.81,12.76]	0.14	0
**HHV6B**	-0.57 [-8.17,7.03]	0.88	0	2.06 [-4.86,8.97]	0.56	0	2.79 [-4.17,9.75]	0.43	0
**HHV7**	-2.97 [-11.6,5.66]	0.5	0	0.52 [-7.39,8.43]	0.9	0	2.02 [-7.25,11.29]	0.67	11

Supplementary Tables

**KSHV**	26.72 [-3.35,56.79]	0.08	37	14.61 [-7.55,36.77]	0.2	19	16.14 [-8.48,40.77]	0.2	28
**HPV16**	19.04 [1.75,36.32]	0.03	0	**20.48 [4.77,36.18]**	**0.01**	0	**20.78 [5.03,36.53]**	**0.01**	0
**HPV18**	6.71 [-13.74,27.16]	0.52	9	8.48 [-10.49,27.46]	0.38	10	9.93 [-7.48,27.33]	0.26	0
**BK**	-20.19 [-55.61,15.23]	0.26	82	-11.77 [-30.99,7.45]	0.23	49	-10.03 [-30.32,10.26] †	0.33	53
**JC**	-2.26 [-18.74,14.22]	0.79	78	-3 [-15.5,9.5]	0.64	66	-3.64 [-16.18,8.9]	0.57	67
**MCV**	-6.12 [-22.35,10.12]	0.46	75	-7.42 [-21.24,6.4]	0.29	70	-6.58 [-20.09,6.93]	0.34	69
**Ct**	-4.5 [-25.19,16.18]	0.67	78	-3.39 [-15.33,8.54]	0.58	43	-4.07 [-17.81,9.67]	0.56	55
**Tg**	10.23 [-1.74,22.21]	0.09	44	3.67 [-4.19,11.52]	0.36	0	3.04 [-4.84,10.92]	0.45	0
**Hp**	6.86 [-1.06,14.79]	0.09	0	2.76 [-4.58,10.09]	0.46	0	1.1 [-6.33,8.52]	0.77	0
**PBI**	2.88 [-3.54,9.29]	0.38	76	0.96 [-3.42,5.34]	0.67	53	0.82 [-3.81,5.44]	0.73	57
**Neuro PBI**	3.86 [0.74,6.99]	**0.02**	32	1.58 [-0.75,3.91]	0.18	0	1.41 [-0.93,3.76]	0.24	0

Abbreviations: BK=BK virus; CMV=Cytomegalovirus; Ct=*C.trachomatis*; EBV=Epstein-Barr virus; HHV=Human herpesvirus; Hp=*H.pylori*; HPV=Human Papillomavirus; HSV=Herpes simplex virus; JC=John Cunningham virus; KSHV=Kaposi's sarcoma-associated herpesviruses; MCV=Merkel Cell virus; PBI=Pathogen burden index; Tg=*T.gondii*; VZV=Varicella zoster virus. The neuro PBI included the nine herpesviruses, JC virus, and *T.gondii*.

Supplementary Tables

Supplementary Table 5. Meta-analysis results for APOE e2 and e4 and pathogen serostatus (and burden) interactions and neuroimaging outcomes. P values are presented and full results are available on request. Associations significant at p<0.05 are in bold, and no associations survived multiple testing correction. Model 1 included adjustments for total intracranial volume and other technical covariates, and 10 genetic principal components; model 2 additionally for age and sex; and model 3 additionally for BMI, smoking status, educational attainment, socioeconomic position, and alcohol intake.

		Brain volume			Hippocampal volume			White matter lesion volume		
		Model 1	Model 2	Model 3	Model 1	Model 2	Model 3	Model 1	Model 2	Model 3
		p	p	p	p	p	p	p	p	p
Serostatus										
**HSV1†***	*APOE* e2	0.94	0.74	0.82	0.34	0.35	0.29	0.87	0.71	0.72
	APOE e4	0.28	0.47	0.46	0.25	0.34	0.38	0.64	0.48	0.44
**HSV2†**	*APOE* e2	0.61	0.45	0.39	0.71	0.77	0.79	0.26	0.3	0.27
	APOE e4	0.62	0.81	0.74	0.73	0.63	0.6	0.17	0.08	0.09
**VZV†**	*APOE* e2	0.98	0.87	0.7	0.29	0.28	0.25	0.93	0.9	0.67
	APOE e4	0.94	0.96	0.98	0.58	0.62	0.58	0.93	0.73	0.59
**EBV†**	*APOE* e2	0.65	0.71	0.85	0.38	0.64	0.38	0.16	0.55	0.59
	APOE e4	0.69	0.99	0.95	0.47	0.72	0.83	0.76	0.68	0.61
**CMV†***	*APOE* e2	0.82	0.35	0.34	0.99	0.92	0.8	0.25	0.14	0.15
	APOE e4	0.16	0.41	0.26	0.79	0.98	0.96	0.4	0.37	0.38
**HHV6A†**	*APOE* e2	0.83	0.98	0.98	0.74	0.83	0.71	0.34	0.4	0.5
	APOE e4	0.42	0.87	1	0.62	1	0.9	0.12	0.6	0.53
**HHV6B†**	*APOE* e2	0.69	0.62	0.68	0.45	0.34	0.35	0.65	0.76	0.74
	APOE e4	0.83	0.38	0.47	0.9	0.96	0.81	0.37	0.54	0.81
**HHV7†**	*APOE* e2	0.69	0.37	0.52	0.58	0.52	0.52	0.84	0.63	0.63
	APOE e4	0.47	0.84	0.47	0.52	0.33	0.35	0.96	0.74	0.77
**KSHV†***	*APOE* e2	0.55	0.71	0.87	0.33	0.34	0.36	0.73	0.86	0.73
	APOE e4	0.76	0.59	0.44	0.37	0.63	0.6	0.54	0.61	0.5
**HPV16***	*APOE* e2	0.07	0.75	0.97	0.21	0.5	0.41	0.15	0.45	0.55
	APOE e4	0.8	0.54	0.29	0.81	0.81	0.75	0.33	0.59	0.44
**HPV18***	*APOE* e2	0.76	0.35	0.45	0.38	0.25	0.27	0.28	0.26	0.28
	APOE e4	0.36	0.49	0.23	0.32	0.32	0.21	0.59	0.65	0.72
**BK**	*APOE* e2	0.79	0.98	0.75	0.74	0.84	0.68	0.45	0.46	0.56
	APOE e4	0.63	0.63	0.29	0.93	0.9	0.97	0.88	0.59	0.68

Supplementary Tables

**JC†**	*APOE* e2	0.44	0.72	0.91	0.19	0.25	0.26	0.29	0.44	0.37
	APOE e4	0.85	0.6	0.6	0.45	0.24	0.22	0.52	0.3	0.25
**MCV**	*APOE* e2	0.31	0.61	0.56	0.09	0.08	**0.04**	0.06	0.18	0.17
	APOE e4	0.5	0.34	0.28	0.79	0.6	0.51	0.39	0.55	0.55
**Ct**	*APOE* e2	0.99	0.98	0.75	0.38	0.34	0.31	0.32	0.26	0.28
	APOE e4	0.35	0.37	0.23	0.76	0.53	0.58	0.88	0.82	0.74
**Tg†**	*APOE* e2	0.42	0.93	0.85	**0.003**	**0.004**	**0.004**	0.14	0.13	0.1
	APOE e4	0.86	0.49	0.38	0.44	0.28	0.27	0.94	0.69	0.83
**Hp**	*APOE* e2	0.76	0.94	0.77	0.29	0.23	0.21	0.36	0.45	0.44
	APOE e4	0.27	0.47	0.49	0.96	0.87	0.71	0.59	0.41	0.3
Pathogen burden										
**PBI**	*APOE* e2	0.56	0.66	0.62	**0.02**	**0.01**	**0.004**	0.07	0.07	0.07
	APOE e4	0.71	0.72	0.74	0.81	0.69	0.62	0.75	0.91	0.94
**Neurotropic PBI**	*APOE* e2	0.44	0.49	0.54	**0.03**	**0.03**	**0.01**	0.13	0.1	0.1
	APOE e4	0.5	0.93	0.98	0.82	0.58	0.65	0.76	0.93	0.8

Abbreviations: BK=BK virus; CMV=Cytomegalovirus; Ct=*C.trachomatis*; EBV=Epstein-Barr virus; HHV=Human herpesvirus; Hp=*H.pylori*; HPV=Human papillomavirus; HSV=Herpes-simplex virus; JC=John Cunningham virus; KSHV=Kaposi's sarcoma-associated herpesviruses; MCV=Merkel Cell virus; PBI=Pathogen burden index; Tg=*T.gondii*; VZV=Varicella zoster virus. †= pathogen considered to be neurotropic .*analyses did not run in all studies due to insufficient degrees of freedom.

Supplementary Tables

Supplementary Table 6. Meta-analysis results for associations of Merkel Cell virus, *T.gondii*, and pathogen burden indices with hippocampal volume, stratified by APOE e2 carrier status.

Estimates, 95% confidence intervals, and p values are presented. For pathogen burden exposures, estimates represent the change in outcome per 0.1 unit increase in burden index. Associations significant at p<0.05 are in bold. Model 1 included adjustments for total intracranial volume and other technical covariates; model 2 additionally for age and sex; and model 3 additionally for BMI, smoking status, educational attainment, socioeconomic position, and alcohol intake.

		Hippocampal volume					
		Model 1		Model 2		Model 3	
		β[95%CI] (ml)	p	β[95%CI] (ml)	p	β[95%CI] (ml)	p
**Merkel Cell virus**	*APOE* e2 carrier	-0.2 [-0.43,0.02]	0.08	-0.17 [-0.41,0.06]	0.15	-0.23 [-0.47,0.01]	0.06
	APOE e2 non-carrier	0.05 [0,0.11]	0.06	0.05 [0,0.1]	0.07	0.05 [0,0.1]	0.07
T.gondii	*APOE* e2 carrier	**-0.22 [-0.36,-0.07]**	**0.003**	**-0.18 [-0.32,-0.03]**	**0.02**	-0.15 [-0.32,0.02]	0.08
	APOE e2 non-carrier	-0.03 [-0.09,0.03]	0.38	0 [-0.06,0.06]	0.99	-0.01 [-0.07,0.05]	0.81
**Pathogen burden index**	*APOE* e2 carrier	**-0.09 [-0.15,-0.02]**	**0.01**	**-0.07 [-0.13,-0.02]**	**0.009**	**-0.07 [-0.13,-0.01]**	**0.01**
	APOE e2 non-carrier	0 [-0.02,0.03]	0.69	0.01 [-0.01,0.03]	0.41	0.01 [-0.01,0.03]	0.45
**Neurotropic pathogen burden index**	*APOE* e2 carrier	**-0.05 [-0.09,-0.01]**	**0.01**	**-0.05 [-0.09,0]**	**0.03**	-0.04 [-0.09,0]	0.06
	APOE e2 non-carrier	-0.01 [-0.03,0.01]	0.32	0 [-0.02,0.01]	0.74	0 [-0.02,0.02]	0.79

Supplementary Tables

Supplementary Table 7. Meta-analysed associations of seroreactivity tertiles with brain volume.
Estimates, 95% confidence intervals, p values, and I^2 statistics are presented. Associations significant at $p<0.05$ are in bold, and those significant following multiple testing correction are further indicated by an asterisk. Model 1 included adjustments for total intracranial volume and other technical covariates; model 2 additionally for age, sex, and ethnicity; and model 3 additionally for BMI, smoking status, educational attainment, socioeconomic position, and alcohol intake.

	Brain volume								
	Model 1			Model 2			Model 3		
	β [95% CI]	p	I^2	β [95% CI]	p	I^2	β [95% CI]	p	I^2
**HSV1**	-0.37 [-2.33,1.6]	0.71	0	0.12 [-1.58,1.81]	0.89	0	0.18 [-1.52,1.87]	0.84	0
**HSV2**	-0.85 [-5.95,4.25]	0.74	0	-1.58 [-5.7,2.54]	0.45	0	-0.85 [-5.1,3.41]	0.7	0
**VZV**	-1.43 [-3.3,0.43]	0.13	0	-1.1 [-2.67,0.47]	0.17	0	-1.18 [-2.74,0.39]	0.14	0
**EBV**	-1.09 [-5.25,3.07]	0.61	81	-0.41 [-1.84,1.03]	0.58	0	-0.31 [-1.73,1.12]	0.67	0
**CMV**	-0.32 [-4.23,3.6]	0.87	67	-0.62 [-2.5,1.25]	0.52	12	-0.55 [-2.23,1.12]	0.52	0
**HHV6A**	1.37 [-2.16,4.9]	0.45	42	0.71 [-1.42,2.83]	0.52	0	0.77 [-1.68,3.21]	0.54	18
**HHV6B**	1.32 [-1,3.65]	0.26	0	-1.02 [-2.93,0.88]	0.29	0	-1.12 [-3.01,0.78]	0.25	0
**HHV7**	-1.17 [-3.24,0.91]	0.27	0	-1.62 [-3.35,0.12]	0.07	0	-1.65 [-3.38,0.07]	0.06	0
**BK**	0.92 [-0.78,2.63]	0.29	0	-0.45 [-1.9,1]	0.54	0	-0.59 [-2.04,0.85]	0.42	0

Supplementary Tables

**JC**	-1.47<br>[-4.77,1.82]	0.38	47	-0.37<br>[-3.37,2.63]	0.81	54	-0.63<br>[-4.33,3.07]	0.74	69
**MCV**	-3.54<br>[-7.71,0.64]	0.1	73	-3.52<br>[-8.45,1.41]	0.16	86	-3.02<br>[-7.93,1.88]	0.23	85
**Ct**	1.05<br>[-5.5,7.59]	0.75	63	-0.14<br>[-4.81,4.52]	0.95	46	1.2<br>[-4.64,7.04]	0.69	60
**Tg**	-4.46<br>[-9.68,0.77]	0.09	0	**-6.63**<br>**[-11.26,-2]**	**0.005**	12	**-7.11**<br>**[-14.13,-0.08]**	**0.05**	50
**Hp**	2.89<br>[-0.21,5.99]	0.07	0	1.86<br>[-0.84,4.55]	0.18	0	1.84<br>[-0.9,4.59]	0.19	0

Abbreviations: BK=BK virus; CMV=Cytomegalovirus; Ct=*C.trachomatis*; EBV=Epstein-Barr virus; HHV=Human herpesvirus; Hp=*H.pylori*; HSV=Herpes simplex virus; JC=John Cunningham virus; MCV=Merkel Cell virus; Tg=*T.gondii*; VZV=Varicella zoster virus. Kaposi's sarcoma-associated herpesviruses and Human Papillomavirus 16 and 18 were not tested as seroprevalence were <5% in at least one cohort.

Supplementary Tables

Supplementary Table 8. Meta-analysed associations of seroreactivity tertiles with hippocampal volume.
Estimates, 95% confidence intervals, p values, and I^2 statistics are presented. Associations significant at $p<0.05$ are in bold, and those significant following multiple testing correction are further indicated by an asterisk. Model 1 included adjustments for total intracranial volume and other technical covariates; model 2 additionally for age, sex, and ethnicity; and model 3 additionally for BMI, smoking status, educational attainment, socioeconomic position, and alcohol intake.

	Hippocampal volume								
	Model 1			Model 2			Model 3		
	β [95% CI]	p	I^2	β [95% CI]	p	I^2	β [95% CI]	p	I^2
**HSV1**	0 [-0.05,0.04]	0.9	0	-0.02 [-0.07,0.02]	0.28	0	-0.02 [-0.07,0.02]	0.28	0
**HSV2**	0 [-0.1,0.11]	0.96	7	0.02 [-0.1,0.14]	0.73	14	0.01 [-0.15,0.16]	0.92	34
**VZV**	-0.05 [-0.11,0.02]	0.18	62	-0.05 [-0.11,0]	0.063	45	-0.05 [-0.11,0.01]	0.07	47
**EBV**	-0.02 [-0.06,0.01]	0.19	0	0 [-0.04,0.03]	0.94	0	0 [-0.04,0.03]	0.87	0
**CMV**	-0.02 [-0.06,0.03]	0.44	0	-0.01 [-0.05,0.04]	0.78	0	-0.01 [-0.05,0.03]	0.72	0
**HHV6A**	0.01 [-0.05,0.06]	0.81	0	0.01 [-0.05,0.06]	0.83	0	0 [-0.05,0.06]	0.88	0
**HHV6B**	**0.06 [0.01,0.11]**	**0.02**	2	0.04 [0,0.09]	0.07	0	**0.05 [0,0.1]**	**0.04**	0
**HHV7**	-0.04 [-0.09,0]	0.06	0	-0.04 [-0.08,0.01]	0.09	0	-0.04 [-0.08,0.01]	0.09	0
**BK**	0 [-0.04,0.04]	0.99	2	0 [-0.04,0.03]	0.95	0	0 [-0.04,0.03]	0.87	0

Supplementary Tables

**JC**	-0.02 [-0.06,0.03]	0.51	0	-0.02 [-0.07,0.02]	0.36	0	-0.02 [-0.06,0.03]	0.45	0
**MCV**	-0.03 [-0.07,0.02]	0.23	0	-0.01 [-0.06,0.03]	0.49	0	-0.02 [-0.06,0.03]	0.45	0
**Ct**	0.05 [-0.02,0.11]	0.17	6	**0.07 [0.01,0.13]**	**0.03**	0	**0.07 [0.01,0.14]**	**0.02**	0
**Tg**	0.06 [-0.09,0.21]	0.44	37	0.03 [-0.09,0.15]	0.59	6	0 [-0.14,0.13]	0.96	5
**Hp**	0.05 [-0.03,0.12]	0.22	0	0.03 [-0.04,0.11]	0.36	0	0.03 [-0.05,0.1]	0.51	0

Abbreviations: BK=BK virus; CMV=Cytomegalovirus; Ct=*C.trachomatis*; EBV=Epstein-Barr virus; HHV=Human herpesvirus; Hp=*H.pylori*; HSV=Herpes simplex virus; JC=John Cunningham virus; MCV=Merkel Cell virus; Tg=*T.gondii*; VZV=Varicella zoster virus. Kaposi's sarcoma-associated herpesviruses and Human Papillomavirus 16 and 18 were not tested as seroprevalence were <5% in at least one cohort.

Supplementary Tables

Supplementary Table 9. Meta-analysed associations of seroreactivity tertiles with white matter lesion volume.
Estimates, 95% confidence intervals, p values, and I^2 statistics are presented. Associations significant at p<0.05 are in bold, and those significant following multiple testing correction are further indicated by an asterisk. Model 1 included adjustments for total intracranial volume and other technical covariates; model 2 additionally for age, sex, and ethnicity; and model 3 additionally for BMI, smoking status, educational attainment, socioeconomic position, and alcohol intake.

	**White matter lesion volume**								
	**Model 1**			**Model 2**			**Model 3**		
	% change [95%CI]	p	I^2	% change [95%CI]	p	I^2	% change [95%CI]	p	I^2
**HSV1**	-10.61 [-19.81,-1.41]	0.02	27	-7.19 [-13.99,-0.39]	**0.04**	0	-6.01 [-12.83,0.82]	0.08	0
**HSV2**	-1.9 [-21.52,17.73]	0.85	0	-0.41 [-18.24,17.43]	0.96	0	0.56 [-18.21,19.32]	0.95	0
**VZV**	2.48 [-7.71,12.67]	0.63	48	3.7 [-3.67,11.07]	0.32	19	4.53 [-5.93,14.99]	0.4	56
**EBV**	12.12 [5.67,18.57]	**2e-4**	0	4.58 [-1.36,10.52]	0.13	0	4.44 [-1.53,10.4]	0.15	0
**CMV**	8.08 [0.37,15.79]	**0.04**	0	4.46 [-2.75,11.66]	0.23	0	5.05 [-2.21,12.3]	0.17	0
**HHV6A**	0.14 [-9.57,9.84]	0.98	0	1.82 [-7.02,10.66]	0.69	0	1.99 [-6.99,10.98]	0.66	0
**HHV6B**	-4 [-12.84,4.84]	0.38	5	0.33 [-7.7,8.36]	0.94	4	0.18 [-7.71,8.07]	0.96	0
**HHV7**	7.44 [-0.12,15.01]	0.05	0	5.52 [-1.46,12.49]	0.12	0	5.37 [-1.65,12.39]	0.13	0

Supplementary Tables

**BK**	-5.93 [-12.38,0.51]	0.07	0	-5.67 [-11.61,0.28]	0.06	0	-6.1 [-12.08,-0.11]	**0.05**	0
**JC**	1.53 [-6.9,9.96]	0.72	7	3.78 [-3.76,11.31]	0.33	3	1.95 [-9.23,13.13]	0.73	49
**MCV**	5.15 [-2.48,12.78]	0.19	0	1.56 [-5.41,8.54]	0.66	0	1.52 [-5.52,8.55]	0.67	0
**Ct**	7.12 [-11.39,25.63]	0.45	41	5.33 [-5.33,15.99]	0.33	0	6.87 [-4.04,17.78]	0.22	0
**Tg**	6.71 [-17.88,31.31]	0.59	36	5.58 [-11.46,22.61]	0.52	0	4.63 [-13.49,22.75]	0.62	0
**Hp**	14.01 [-25.93,53.95]	0.49	80	14.71 [-23.7,53.12]	0.45	81	9.36 [-26.28,44.99]	0.61	76

Abbreviations: BK=BK virus; CMV=Cytomegalovirus; Ct=*C.trachomatis*; EBV=Epstein-Barr virus; HHV=Human herpesvirus; Hp=*H.pylori*; HSV=Herpes simplex virus; JC=John Cunningham virus; MCV=Merkel Cell virus; Tg=*T.gondii*; VZV=Varicella zoster virus. Kaposi's sarcoma-associated herpesviruses and Human Papillomavirus 16 and 18 were not tested as seroprevalence were <5% in at least one cohort.
